# Hygienic practice during complementary food preparation and associated factors among mothers of children aged 6–24 months in Debark town, northwest Ethiopia, 2021: An overlooked opportunity in the nutrition and health sectors

**DOI:** 10.1371/journal.pone.0275730

**Published:** 2022-12-09

**Authors:** Agerie Mengistie Zeleke, Gashaw Melkie Bayeh, Zelalem Nigussie Azene

**Affiliations:** 1 Department of Midwifery, School of Public Health, Teda Health Science College, Gondar, Ethiopia; 2 Department of Environmental Health, School of Public Health, Teda Health Science College, Gondar, Ethiopia; 3 Department of Women’s and Family Health, School of Midwifery, College of Medicine and Health Sciences, University of Gondar, Gondar, Ethiopia; National Research Centre, EGYPT

## Abstract

**Background:**

Hygienic practices during complementary food preparation are suboptimal in developing countries, in Ethiopia in particular. Hygienic complementary food preparation is crucial to prevent childhood communicable diseases like diarrhea and associated malnutrition among children aged 6–24 months. However, in Ethiopia, there is a paucity of evidence on the practice of hygiene during complementary food preparation. Thus, this study is aimed to assess the hygienic practice of complementary food preparation and associated factors among women having children aged 6–24 months in Debark town, northwest Ethiopia.

**Methods:**

A community-based cross-sectional study was conducted among 423 mothers with 6–24 months of age children from December 1 to January 30, 2021. A simple random sampling technique was used to select the study participants. Data were collected using an interviewer-administered structured questionnaire. Epi-data version 4.6 and SPSS version 23 software were used for data entry and analysis, respectively. Binary logistic regressions (Bivariable and multivariable) were performed to identify statistically significant variables. Adjusted odds ratio with 95% CI was used to declare statistically significant variables on the basis of p-value < 0.05 in the multivariable logistic regression model.

**Results:**

The study revealed that 44.9% (95% CI (40.2, 49.4%)) of the mothers having children aged 6–24 months had good practice of complementary food preparation. Maternal age of 25–29 years[AOR:3.23, 95% CI: (1.555–9.031)], husband’s attained secondary school and above (AOR:2.65, 95% CI (1.211–5.783)], using modern stove for cooking [AOR:3.33,95% CI (1.404–7.874)], having a separate kitchen[AOR: 8.59, 95%Cl: (2.084–35.376], and having a three bowl dishwashing system(AOR: 8.45, 95% CL: (4.444–16.053)) were significantly associated with good hygiene practice of complementary food preparation.

**Conclusions:**

The findings have indicated that the majority of the mothers had poor hygienic practices of complementary food preparation. Mother’s age, husband’s educational status, type of stove used for cooking, having a separate kitchen, having a three bowl dishwashing system were factors that significantly influenced the hygiene practice of mothers during complementary food preparation. Therefore, training and counseling mothers and caregivers on complementary food processing and preparation is important and such endeavors which inform the development and implementation of complementary food hygiene interventions in urban communities are recommended.

## Background

The World Health Organization (WHO) defines Complementary Feeding(CF) as a process that starts when breast milk alone is no longer sufficient food and liquid to meet the nutritional requirements of the infant [1,2]. Guidelines of infant and young child feeding indicators recommended that complementary feeding should start after 6 months of age with continued breastfeeding up to 24 months or beyond in normal conditions and it should be hygienically prepared, stored, and fed with clean hands using clean utensils, but not bottles and teats [3,4]. This period is a transition from exclusive breastfeeding to family foods. Besides, it is a very critical time in which poor hygienic practice in preparing CF for many young children is predominant which in turn contributes significantly to the high prevalence of gastrointestinal and respiratory illnesses amongst infants [5]. Gastrointestinal diseases associated with preventable food-borne bacteria inflicting children less than two years of age remain a global health challenge [6]. Poor hygienic practices during CF preparation for infants and children play a major role in the occurrence of childhood diarrheal diseases[7]. Studies have shown that diarrheal incidence increases at the age when complementary foods are usually introduced as unhygienic preparation and handling of foods can be a source of diarrheal pathogens [8–10]. Diarrhea is obviously associated with malabsorption of significant nutrients, fluid losses and reduced appetite [11] which results in severe childhood nutritional problems such as wasting, and stunting [12]. Although hardware components such as improved water supplies and sanitation facilities make it easier to practice it, better hygiene still makes a huge difference in health especially in keeping children safe from infection caused by feeding of contaminated foods [13]. Improving hygiene during complementary feeding is given a special attention in the Sustainable Under nutrition Reduction in the world (SURE) program and multi-sectoral intervention are being carried out to achieve the purpose [14].

Worldwide, the lives of approximately 525,000 children are lost each year from 1.7 billion cases of different infectious diseases like childhood diarrhea with the highest mortality rates reported among children aged less than 2 years in south Asia and sub-Saharan Africa [15]. Furthermore, 230,000 children die every year globally because of diarrheal diseases associated with complementary food contaminations [3]. In African countries, data indicated that food could be more important than water in transmitting diarrheal disease, and it is estimated that 30% - 40% of children aged less than 5 years suffer from different microbial pathogen diseases [5,16]. This corresponds with reports that claim that at least 70% of diarrhea-related pathogens among children are caused by contaminated complementary food [17]. Scientific evidence indicates that poor hygiene complementary feeding practices can have profound consequences on the growth, development, and survival of infants and children [16,18]. This is explained by a study conducted in Malawi which stated that 27% of 6–24 months old children were reported to have had diarrhea in the 2 weeks after initiation of CF that resulted in 80% of the children suffering from reduced height and growth rate while the rest 20% suffered from underweight [19]. Across the three studies conducted in rural India, it is shown that the prevalence of child stunting ranged from 25% to 50% [20]. According to the 2019 mini Demographic and Health Survey of Ethiopia (EDHS), current prevalence of CF is 13%, in addition to breast milk. On the other hand, infant mortality was 43% caused by preventable bacterial pathogens provoking diseases. Inadequate food hygiene is considered to be one of the major contributors to diarrhea [21]. Appropriate CF requires good hygiene while preparing complementary foods, availability of sufficient house hold-level food, and adequate nutritional knowledge application by caregivers [22]. Lack of understanding of the risk of hygiene practices of complementary feeding is the major concern in preventing and controlling food-borne diseases inflicted on 6–24 months old children in Ethiopia [23–25]. Improving food hygiene practices play a great role in reducing child morbidity and mortality. Previous some studies were focused on the prevalence and practice of complementary food, however, hygienic practice during complementary food preparation and some interesting variables were not addressed in Ethiopia, specifically study setting. On top of this, there is a problem in real hygiene practices of complementary feeding, resulting in serious consequences of poor child health outcomes. Therefore, the present study is aimed to assess the hygienic practice and associated factors of complementary food preparation among mothers of children aged 6–24 months in Debark town, northwest, Ethiopia.

## Materials and methods

### Ethical considerations

Ethical approval was obtained from the Ethical Review Committee of Amhara Public Health Institution (APHI) with ethical letter protocol number: ref No/APHI/1499/2021 After an official letter had been submitted to Debark town health department’s office, permission letters were collected/ obtained from debark town health department’s office. After explaining the purpose of the study, verbal informed consent was obtained from each study participants(mothers//guardians). The respondents were also informed that they have the full right to withdraw or refuse at any time from the process. Confidentiality of information given by each respondent was kept properly and anonymity was explained clearly for the participants.

### Study area

The study was conducted in Debark town, Amhara regional state, northwest Ethiopia. The town is 828 km far from Addis Ababa, the capital of Ethiopia, and about 260 kilometers from Bahir Dar city, the capital of Amhara regional state. The town has three Kebeles (the smallest administrative units of Ethiopia) and a total population of 25,350. The town has now 1 hospital, 1 health center, 2 medium clinics, 4 primary clinics, 6 pharmacies, and 2 health posts providing maternal and child health services.

### Study design and period

A community-based cross-sectional study was employed from December 1 to January 30, 2021.

#### Participants

The source population were all mothers were who have children aged from 6 to 24 months and resides at least six month in the debark town, while all mothers with children aged from 6 to 24 months old and who present in each kebele during the data collection period were study population.

#### Eligibility criteria

All selected mothers that started complementary food for children aged 6–24 months, or guardians who lived in the town for a minimum of six months during the data collection period were included. Mothers/guardians who were unable to respond, seriously ill, and unable to hear were excluded from the study.

#### Study variables and measurements

The outcome variable of this study was the hygienic practice in complementary food preparation, while others like socio-demographic factors, knowledge of critical time for hand washing, and environmental-related factors, knowledge and attitude of mothers towards hygiene during complementary food preparation were the explanatory variables.

The hygienic practice of complementary food preparation was assessed based on a related sixteen items questionnaire (Cronbach’s alpha 0.78) and supported by observational questions which contained a three-points score scale (always, sometimes and never) and some categorical questions (yes and no),which were used for the analysis of the responses. Accordingly, the mean of the responses was computed. Participants who scored of ≥50% and above of the practice measuring items about hygienic complementary food preparation were labeled as having good hygienic practice and score of <50% in complementary food preparation as a poor hygienic practice [26]. Knowledge of mothers about the hygiene of complementary food preparation was assessed based on related twenty questions (Cronbach’s alpha 0.73), and a three-point score scale was used for the analysis of responses (yes always, yes sometimes, and never). Those study participants who scored mean and above mean of the sum of the knowledge questions were considered as having good knowledge [25]. Respondents were asked to respond to the 21 attitudinal questions about the hygienic practice during complementary food preparation, each question containing a 5 points likert scale (1-strongly agree, 2-agree, 3-not applicable/undecided 4-disagree and 5-strongly disagree). The responses were dichotomized as desirable and none desirable attitude, and then composited. Also, the study participants who scored mean and above mean of the attitude questions were considered as holding the desirable attitude.

#### Sample size determination

The sample size was determined using a single population proportion formula. Accordingly, the formula for sample size determination uses is: n = (Zα/2)^2^ **x** [(p1q1)/(d)^2^], the following assumptions were considered: n denotes the sample size, Zα/2 is the reliability coefficient of standard error at 5% level of significance = 1.96, p = the status of the good hygienic practice of complementary food preparation based on the 49.7% score from a previous research conducted in Woldia town, Ethiopia [27]. In that view, 10% non-response rate, and the final sample size approximately was 423.

The sample size was also calculated using factors associated with knowledge and practice towards hygiene by considering the following assumptions; two-sided confidence interval = 95%; power = 80%; ratio (unexposed to exposed) = 1 and 10% non-response rate (NR) (Table 1).

**Table 1:** Sample size for second objective.

Factors	%Outcome in unexposed	%Outcome in exposed	Odds ratio	Calculated sample size	sample size+NR	Reference
Residence	30.96%	80.41%	9.15	38	42	[28]
Access to media	11.04%	66,29%	15.8	20	22	[28]
Presence of handwashing facility	15.69	65.95	10.4	36	57	[28]
Private latrine ownership	11.29%	66.37%	14.1	32	35	[28]
Mothers educational level	38.27	61.76	0.38	120	132	[27]
	38.27	58.09	0.45	158	174	[27]
Place of delivery	73.33	47.67	0.33	218	240	[27]

Therefore, the sample size obtained by using single population formula (423) is higher than the sample size calculated by using the second objective (using factors significantly associated with outcome variable). Therefore, the minimum sample size to represent the source population was 423.

#### Sampling procedures

Simple random sampling technique was used to select study participants. The study participants were selected from three Kebeles using a simple random sampling technique. According to Debark town’s health office report, the total number of mothers of two years old children in the study area was 10,132. The sample, 423 was drawn out from these mothers with children aged between 6 and 24 months old. The number of study participants’ were allocated for each Kebele based on proportion to population size allocation methods. The list of study participants by their name and residence was found from health posts log book. Data collectors went house to house and collect the data through walk through approach randomly. When two and above mothers with 6–24 months old children were found in household, one the mother with 6–24 months old children was selected randomly.

#### Data collection tool and procedure

The questionnaire was developed after intensively reviewing related previous studies [29–31]. It has three parts (socio-demographic, environmental and housing and hygiene practices). It was first prepared in English and translated to Amharic (local language) and again translated back to English by language experts (**AV) S1** and **S2 Annex**. Data were collected by three health extension workers both in a face-to-face interview a using structured questionnaire and supported by observation under the supervision of the investigator (supervisor). The data were then checked for any incompleteness and were later coded.

#### Data quality management

A pre-test was done on 5% of the sample size (30 participants) in Dabat town, Keble one. A- one day training was given for the data collectors before the actual data collection. The training has covered the aim of the study, procedure, inclusion and exclusion criteria, data collection techniques, contents and details of the questionnaire, the art of interviewing and clarification. Moreover, during data collection, the supervisor has checked how the data collection process was going on. At the end of each data collection, the principal investigator also checked the completeness of the filled questionnaires. In other words, every questionnaire was checked before data entry by the principal investigator. Multicollinearity was also checked to see the linear correlation between the independent variables by using a standard error and variance inflation factor. Variables with the standard error of >2 and the variance inflation factor (VIF) from one to ten were checked by the multivariable analysis. Hosmer-Lemeshow goodness of fit test was used to check for model fitness by looking at the cut of point P-value > 0.05. The continuous variables such as age were tested using the normal curve with a histogram.

#### Statistical analysis

Data entry was performed using the statistical program Epi-Data version 4.6 and then exported into SPSS version 23 for analysis. Descriptive statistics were carried out and presented with narration, figure, and tabulation. Binary logistic regression (Bivariable and Multivariable) was performed to identify statistically significant variables. Variables that had *P*-value less than 0.25 in the binary logistic regression analysis was entered to multiple logistic regressions statistical analysis to identify independent associated factors of hygiene practice during complementary food preparation. Adjusted odds ratio with a 95% confidence interval was used to declare statistically significant variables on the basis of p-value <0.05 in the multivariable binary logistic regression model. Hosmer and Lemeshow goodness of fit test was performed and the decision was made at P>0.05.

## Results

### Respondents’ socio-demographic characteristics

Of the overall sample required (N = 423), all participants were included in the study; hence, the response rate was 100%. Almost all (96%) of the study participants were Amhara by ethnicity. The mean age of the respondents was (29.8±5.7sd) years. Besides, the majority of the respondents (377(89.1%) were married (Table 2).

**Table 2:** Bivariate analysis of the association of socio-demographic factors with hygiene practice during complementary food preparation among mothers with 6–24 months aged of children in Debark town, Ethiopia, 2021.

Variables	Frequency (%)	Hygiene practice		COR((95% CI)	p-value
		Good	Poor		
**Age groups**					
19–24	86(20.3)	47	39	0.79(0.385,1.618)	0.519
25–29	187(44.2)	57	130	0.29(0.873,2.763)	0.000
30–34	102(24.1)	57	45	0.83(1.217,3.432)	0.601
≥35	48(11.3)	29	19	1.00	
**Ethnicity**					
Amhara	406(96.0)	180	226	0.27(0.23–2.08)	0.499
Tigray	13(3.1)	7	6	0.38(0.03–2.57)	0.253
Qimant	4(0.9)	3	1	1	1
**Mothers’ marital status**					
Married	377(89.1)	172	205	1.20(0.31–2.24)	0.719
Divorced	29(6.9)	11	18	0.87(0.34–3.89)	0.829
Windowed	17(4.0)	7	10	1	
**Mothers’ educational**					
Secondary & above	110(26.0)	69	41	6.73(3.49–12.99)	0.000
Primary school	59(13.9)	27	32	2.01(1.12–2.29	0.005
Able to read write	169(40.0)	77	92	2.00(1.05–3.79)	0.035
Unable to read write	85(20.1)	17	68	1	
**Husband’s educational**					
Secondary & above	171(40.4)	103	68	4.80(2.23–9.83)	.000
Primary school	118(27.9)	31	87	1.38(0.81–2.33)	0.234
Able to read write	84(19.9)	44	40	4.25(2.55–7.09)	.000
Unable to read write	50(11.8)	12	38	1	0.000
**Mothers’ occupation**					
House wife	299(70.7)	111	188	0.61(0.08–0.31)	.075
Employed	61(14.4)	48	13	3.81(0.35–1.05)	.001
Merchant	63(14.9)	31	32	1	
**Husband’s occupation**					
Farmer	130(30.7)	53	77	1.36(0.43–1.25)	.256
Employed	136(32.1)	86	50	3.38(2.26–12.03)	.000
Daily worker	47(11.1.1)	14	33	0.84(0.57–2.50)	.637
Merchant		37	73	1	
**Annually family income(Ethiopian Birr)**					
≥2400	222(52.5)	118	104	2.03(1.38–3.01)	.000
<2400	201(47.5)	72	129	1	
**Family size**					
<5	250(59.1)	113	137	1.03(0.70–1.52)	.888
≥5	173(40.9)	77	96	1	

### Environmental and housing related characteristics

Three hundred and nineteen (51.8%) of the study participants had access to information from media about hygienic complementary food preparations. In relation to the hygiene practice training, 353(83.5%) of the study participants had got food handling-related training. In the case of latrine, 363(85.8%) of the households had a latrine, of those one hundred and seventy–seven (41.8%) of the participants had slaved type of pit latrine. Only 27(6.7%) of the study participants had hand washing facility with soap or detergent and sufficient water near their latrines.

More than half (223 (52.7%)) of the study participants did not wash their hands with soap after cleaning their children’s buttocks. In the present study, 55.3% of the study participants had a separated three bowls dishwashing system kitchen facility (**Table 3)**.

**Table 3:** Bivariate analysis of the association of environmental and housing related factors with hygiene practice in complementary food preparation among mothers with 6–24 months aged of children in Debark town northwest Ethiopia, 2021(n = 423).

Variables	Frequency (%)	Hygiene practice		COR((95% CI)	p-value
		Good	Poor		
**Access to media**					
Yes	219(51.8)	116	103	1.98(1.34–2.92)	0.001
No	204(48.2)	74	130	1	
**Training took about food preparation**					
Yes	70(16.5)	33	37	1.11(0.67–1.86)	0.682
No	353(83.5)	157	196	1	
**Presence of latrine**					
Yes	263(85.5)	164	199	1.08(0.54–1.61)	0.790
No	60(14.2)	26	34	1	
**Type of latrine in household**					
Pour flush and well ventilated	115(27.2)	58	57	1.55(0.93–2.56)	0.335
Pit with slab	177(41.8)	80	97	1.25(0.77–1.96)	0.092
Pit without slab/open	131(31.0)	52	79	1	
**Latrine availability for single household (n = 363**)					
Yes	197(54.3)	87	110	0.91(0.72–1.66)	0.672
No	166(45.7)	77	89	1	
**Have hand wash facility with near to latrine**					
Yes	27(6.4)	19	8	3.13(1.34–7.31)	0.009
No	336(93.6)	171	225	1	
**Hand wash after visiting toilet**					
Yes	274(64.8)	117	157	0.78(0.52–1.16)	0.125
No	149(35.2)	73	76		
**Hand wash with soap after child’s bottom**					
Yes	200(47.3)	126	74	4.23(2.81–6.36)	.000
No	223(52.7)	64	159	1	
**Child’s hand wash with soap after defecation**					
Yes	223(52.7)	142	81	5.55(3.63–8.48)	.000
No	200(37.3)	48	152	1	
**Three bowl system dish washing facility**					
Yes	189(44.7)	147	42	15.55(9.65–25.04)	.000
No	234(55.3)	63	191	1	
**Separated kitchen for food preparation**					
No	50 (34.3)	3	47	1.00	.000
Yes	373(65.7)	187	186	15.75(4.817,51.499)	
**Type stove for cooking**					
Traditional	276(65.2)	136	140	1	
Modern	147(34.8)	54	93	1.69(1.11–52)	.014
**Sources of water**					
Piped water	266	135	131	1.70(1.11–2.62)	.015
Protected	27(6.7)	6	21	0.47(1.41–9.22)	.007
Spring water	130(30.4)	49	81	1	
**Distance from home to water source**					
In the yard	134(31.7)	77	57	1.69(1.23–3.30)	.253
<30 minute	145(34.3)	49	96	0.64(1.05–2.71)	.321
≥30 minute	144(34.0)	64	80	1	
**Water treatment options**					
Treated	54(12.8)	28	26	1.38(0.78–2.44)	.274
No usage of treatment	369(87.2)	162	207	1	

### Maternal knowledge and attitude towards hygienic complementary food preparation

Findings of this study showed that 274(64.8%) of the study participants had a desirable attitude towards hygienic complementary food preparation **(S1 Fig)**.

According to knowledge status, only one hundred and eighty-one (42.8%) of the study participants had good knowledge about hygienic complementary food preparation methods. Among all study participants, the majority (376 (88.9%)) of them believed that raw and cooked complementary foods that were stored together did not lead to complementary food contamination. The study revealed that only ninety-eight (23.2%) of the respondents had unvarnished nails and 131(32.0%) did not wash their hands with water and soap before complementary food preparations *(***Table 4).**

**Table 4:** Bivariate analysis of the association household knowledge with hygiene practice during complementary food preparation with 6–24 months aged of children in Debark town northwest Ethiopia, 2021(n = 423).

Variables	Frequency (%)	Hygiene practice		COR((95% CI)	p-value
		Good	Poor		
**Hand wash before food preparation**					
Yes	337(79.7)	152	185	0.96(0.60–1.55)	.879
No	86(20.3)	38	48	1	
**Hand wash after change diaper**					
Yes	336(79.4)	167	169	2.75(1.63–4.64)	.000
No	87(20.6)	23	64	1	
**Important child’s hand wash before child feeding**					
Yes	20(4.3)	9	11	1.04(0.41–2.48)	.994
No	403(95.7)	181	222	1	
**Important hand wash before cooking**					
Yes	370(87.5)	177	193	2.82(1.46–5.45)	.002
No	53(12.5)	13	40	1	
**Important hand wash before serving meal**					
Yes	413(97.6)	188	225	3.34(0.70–15.93)	.130
No	10(2.4)	2	8	1	
**Hand wash after visiting toilet**					
Yes	344(81.3)	172	172	3.339(1.92–5.97)	.000
No	79(18.7)	18	61	1	
**Keeping raw and cooked dose no lead food contamination**					
Yes	47(11.1)	14	33	0.48(0.25–0.93)	.030
No	346(88.9)	176	200	1	
**Food handing should be avoided during diahirreal illness**					
Yes	330(78.0)	158	172	1.75(1.08–2.83)	.022
No	93(22.0	32	61	1	
**Hand wash is not important after handing raw beef or poultry**					
Yes	26(6.1)	3	23	0.15(0.4–0.50)	.002
No	397(93.9)	187	210	1	
**Lid container is not import and to store food**					
Yes	19(4.5)	5	14	0.42(0.15–1.20)	.105
No	404(93.5)	185	219	1	
**Leftover foods should be eaten without reheating**					
Yes	23(5.4)	11	12	1.13(0.49–2.63)	.773
No	400(94.6)	179	221	1	
**Contaminated water can be a source of food contamination**					
Yes	342(80.9)	166	176	2.24(1.33–3.78)	.002
No	81(19.1)	24	57	1	
**Fruits and vegetables should not washed before eating**					
Yes	47(11.1)	25	22	1.45(0.79–2.67)	.228
No	376(88.9)	165	211	1	
**Knowledge mean score**					
Poor	242(57.2)	42	34	1	
Good	181(42.8)	148	199	0.60(0.42–0.92)	.015

### Hygienic practice of complementary food preparation

The results of this study showed that the overall prevalence of good practices of complementary food preparation was found to be (44.9% 95% CI 40.2, 49.4%). Of this, participants 403(95.7%) had utensils clean meticulously during complementary food preparation. Two hundred and seventy-two (64.3%) of the study participants washed equipments (utensils) with hot water after feeding their children (Table 5).

**Table 5:** Frequency of each hygiene practice during complementary food preparation with 6–24 months aged of children in Debark town northwest Ethiopia, 2021(n = 423).

Variables	Category	Frequency	(%)
Hand wash before food preparation	Yes No	292 131	69.0 31.0
Hand wash after touched contaminated	Yes No	139 284	32.9 67.1
Utensils meticulously clean	Yes No	403 20	95.7 4.3
Hand wash before child feeding	Yes No	183 240	43.3 56.7
Child’s hand wash before child feeding	Yes No	146 277	34.5 65.5
Child feeding private utensils	Yes No	248 175	58.6 41.4
Clean child bottle feeding	Yes No	101 322	23.1 76.9
Private utensil clean	Yes No	327 96	77.3 22.7
utensils clean with soap	Yes No	235 188	55.6 44.4
Food utensils clean with hot water	Yes No	272 151	64.3 35.7
Separated store cooked and raw food	Yes No	275 148	65.0 35.0
cooked and raw food sore separated in utensil	Yes No	259 164	61.2 38.8
Serve cooked food within 2 hours	Yes No	137 286	32.4 67.6
Stored food properly covered	Yes No	255 168	60.3 39.7
Serve leftover food	Yes No	117 306	72.3 37.7
Short and clean fingernails	Yes No	339 70	92.9 7.1
Hygiene practice (mean score)	Good practice Poor practice	90 233	44.9 55.1

### Factors associated with hygiene practice of complementary food preparation

The association between all potential independent variables and hygienic practice during complementary food preparation was analyzed using binary logistic regression. Accordingly, in the bivariable binary logistic regression analysis, predictor variables such as age, both maternal and husbands’ educational status, occupational status, annually income, knowledge and attitude towards complementary food preparation, household source of water, access to media, having a type of latrine, presence of hand wash facility near their latrine, mothers who cleaned their baby buttock after deification, type of stove used for cooking, having separated kitchen and three bowls for complementary food preparations were explored to significantly influenced the hygienic practice mothers during complementary food preparation. After controlling for confounders in a multivariable binary logistic regression analysis, age, husband’s educational status, having a modern type of stove, having a separate kitchen and having three bowls dishwashing system remained to significantly influence the hygienic practice of complementary food preparation among mothers. Hence, the odds of having good hygienic practice of complementary food preparation among mothers in the age group of 25–29 years old were 3.23 times more likely [AOR:3.23, 95% CI: (1.555–9.031)] than those mothers aged ≥35 years old.

A husband with an educational background of secondary and above was 2.65times more likely to have good complementary food preparation practice [AOR: 2.65, 95% CI (1.211–5.783] as compared to a husband who was unable to read and write able to read and write and had primary school level of education. Study participants who had three bowl dishwashing facility were 8.45 times more likely [AOR: 8.45, 95% CL: (4.444–16.053)] to have good attitude toward hygienic complementary food preparation than those mothers who had not three bowls dishwashing facilities to have good hygienic complementary food preparation.

Mothers who had modern type of cooking stove were 3.33 times more likely to have good hygienic practice of complementary food preparation than those who had no such type of cooking stove [AOR: 3.33, 95% CI: (1.404–7.874)]. Respondents who had separate kitchens from main house had 8.59 times higher chance [AOR: 8.59, 95% CI: (2.084–35.376)] of good hygienic practice than those respondents who had no kitchen facility separated from main house during complementary preparation **(Table 6).**

**Table 6:** Bivariate and multivariable logistic regression analysis of factors associated with hygiene practice during complementary food preparation among mothers with 6–24 months aged of children in Debark town, Ethiopia, 2021.

Variables	Hygiene practice		COR (95% CI)	AOR(95% CI)	P-value
	Good	Poor			
**Mothers age**					
19–24	47	39	0.79(0.385,1.618)	0.91(0.293,2.802)	0.87
25–29	57	130	0.29(0.873,2.763)	3.23(1.155,9.031) *	0.02
30–34	57	45	0.83(1.217,3.432)	1.26(0.439,3.618)	0.67
≥35	29	19	1.00	1.00	
**Mothers education**					
Secondary and above	69	41	6.73(3.49–12.99)	1.89(0.644,5.535)	0.25
Primary	27	32	2.01(1.12–2.29	1.99(0.405,2.493)	0.99
Able to read and write	77	92	2.00(1.05–3.79)	0.95(0.337,2.652)	0.92
Unable to read & write	17	68	1	1.00	
**Husbands education**					
Secondary and above	103	68	4.80(2.23–9.83)	2.65(1.211,5.783) *	0.015
Primary	31	87	1.38(0.81–2.33)	1.28(0.513,3.167)	0.60
Able to read and write	44	40	4.25(2.55–7.09)	1.57(0.505,4.886)	0.44
Unable to read & write	12	38	1	1.00	
**Mothers occupation**					
House wife	111	188	0.61(0.08–0.31)	1.90(0.848,4.243)	0.12
Employed	48	13	3.81(0.35–1.05)	067(0.206,2.182)	0.51
Merchant	31	32	1	1.00	
**Husbands occupation**					
Farmer	53	77	1.36(0.43–1.25)	0.95(0.367,2.444)	0.91
Employed	86	50	3.38(2.26–12.03)	0.57(0.2109,1.522)	0.27
Daily labor	14	33	0.84(0.57–2.50)	1.24(0.409,3.785)	0.56
Merchant	37	73	1.00	1.00	
**Annually family income (ETB)**					
≥24000	118	104	2.03(1.375,3.005)	1.16(0.528,2.532)	0.72
<24000	72	129	1.00	1.00	
**Sources of water**					
Piped water	135	131	1.70(1.11–2.62)	1.44(0.299,6.925)	0.68
Well protected water	6	21	0.47(1.41–9.22)	0.82(0.272,2.357)	0.65
Spring water	49	81	1	1.00	
**Access to media**					
Yes	116	103	1.98(1.340,2.921	0.65(0.246,1.699)	0.37
No	74	130	1.00	1.00	
**Latrine types in household**					
Pour flush latrine	58	57	1.55(0.932,2.5641	0.54(0.203,1.419)	0.21
Pit with slab latrine	80	97	0.23(0.771,1.971)	0.53(155,1.793)	0.310
Pit without slab/open latrine	52	79	1.00	1.00	
**Presence of hand wash facility near to latrine**					
Yes	19	8	3.13(1.336,7.309)	2.53(2.768,8.338)	
No	171	225	1.00	1.00	0.13
**Three bowls dish washing facility**					
Yes	147	42	15.55(9.654,25.036)	8.45(4.444,16.053)**	
No	43	191	1.00	1.00	0.000
**Hand wash after baby’s buttock clean**					
No	64	159	1.00	1.00	
Yes	126	74	4.23(2.813,6.362)	1.66(0.897,3.077)	0.11
**Types of cooke stove**					
Cultural	136	140	1.00	1.00	
Modern	54	93	1.69(1.111–521)	3.33(1.404,7.874)*	0.007
**Separated kitchen for food preparation**					
No	3	47	1.00	1.00	
Yes	187	186	15.75(4.817,51.499)	8.59(2.084,35.376)*	0.003
**Knowledge**					
Poor	42	34	1.00	1.00	
Good	148	199	0.60(1.08,2.738)	1.20(0.628,2.301)	0.57
**Attitude**					
Undesirable	83	66	1.96(1.310,1.940)	1.34(0.747,2.417)	0.34
Desirable	107	167	1.00	1.00	

NB: The Hosmer and Lemeshow model fitness test p-value was 0.821, VIF was 1.06–2.58, Standard err >2.1, 1.00 = Reference category

* = statistically significant at p<0.05

** = statistically significant at p<0.000.

## Discussion

This study assessed the hygienic practice of complementary food preparation among mothers with children aged 6–24 months and was living in Debark town. Similarly, the study showed that the overall prevalence of good hygienic practice of complementary food preparation among mothers was 44.9% (95% CI (40.2, 49.4%)). This finding is nearly higher than previous studies conducted in different regions of Ethiopia: Harar(39.6%) [32] and Bahir Dar (38.9%) [28]. This difference could be due to the study setting, for the previous study was conducted amongst both rural and urban communities; as a result, women might have less access to information about hygienic practice and that might have helped them to have hygienic practice of complementary food preparation [28,32]. The other reason might be the difference in terms of prevalence of food hygiene practice training. In this study,83.5% of the mothers have taken complementary food related trainings which is higher than that of the study conducted in Harar, where 20.6% of the mothers had food handling trainings [32]. However, this figure is lower than the studies conducted in Sudan (52.1%) [33], Abobo district, Ethiopia (65%) [34], and Dangila, Ethiopia (52.5%) [35]. These observed discrepancy might be due to not only the awareness, and level of knowledge about the hygiene in terms of complementary food preparation practice among mothers but also be due to the socio-demographic characteristics and the number of sample size variations. The differences in the practice of hygienic complementary food preparation among mothers or caregivers might be because of the differences in socio-economic status, level of health services which means that health care providers provide details on how to prepare complementary food during postnatal follow up [33]. There was a difference complementary food preparation rates were inflated when compared to the observed data [36]. In terms of living standard among the study settings and the tools used for assessing hand washing practice. For example, the method used in the current study made use of self- reported practice supported by an observation whereas the studies carried out in Sudan [33] and Abobo district, Ethiopia [34]. Findings of the research conducted in the town of Dangila, Ethiopia were based on food handler mothers who had only worked in food and drink establishments’ area. Accordingly, the practice of hygienic complementary food preparation was overestimated [35] and self-reported hygienic.

The current study showed that the age group of 25–29 years, education status of secondary and above of husbands, having a modern type of stove, having a kitchen separated from the main house and having a three bowls dishwashing system for 6–24 months children’s complementary food preparation tools had statistically significant association with good hygienic practice of complementary food preparation. The odds of performing good hygienic practice among mothers who were in the age group of 25–29 years were 3.23 times higher as compared to those whose age group is ≥35 years [AOR:3.23,95% CI(1.555–9.031)]. Findings reported in a research conducted in Kerala [37] also showed that mothers aged >25 years old were better performers. The possible reason might be loss of experience in complementary food preparation as the age of the mother increases. Health education is given on hygiene, feeding and weaning practices and the preparation of child food at school, and the role of different school clubs in personal hygiene and house sanitation is immense. Husbands who have educational background of secondary and above were 2.65 times more likely to perform good hygienic practice of complementary food preparation than those who are unable to read and write [AOR: 2.65, 95% CI(1.211–5.7834)]. In another study conducted in Harari, Ethiopia [32], educational status was identified as a significant factor, harrier Ethiopia. The reason might be that education enables a husband to help to improve knowledge and awareness of how to prepare a hygienic complementary food. Furthermore, this discrepancy could be due to the fact that study participants might have had trainings and different educational status in relation to complementary food preparation practices. In this study, 171 (40.4%) husbands have had secondary and above educational status. Mothers who had three bowls dishwashing facilities in their kitchen rooms were 8.45 times more likely to have good hygiene practices than those who had no three bowls dishwashing facilities in their kitchen rooms for complementary food preparation [AOR: 8.45,CI 95%(4.444–16.053)]. This finding was supported by a USAID guideline which showed that three bowls dishwashing facilities could maintain complementary food hygiene [38]. The possible explanation for this was households with three bowls dishwashing facility may have properly protected the children from contaminated complementary foods by appropriately cleaning feeding bottles, dishes, and utensils as they lack correctly clean or sterilize the utensils. Another factor associated with this study was that mothers who had modern types of stoves of cooking were 3.33 times more likely to have good hygienic complementary food preparation than those mothers who had cultural types of stoves used to prepare complementary food[AOR: 3.33, CI95%(1.404–7.874)]. Households that have modern stoves might properly kill the microorganism that causes contamination of the complementary food during preparations; thus, modern stoves, beliefs, and safe preparation, and proper storage of complementary foods are the important determinants of hygienic practices during complementary food preparation [39]. In the current study, it was indicated that mothers who had separate kitchens were 8.59 times more likely to have good hygiene during complementary food preparation than those mothers who had no separate kitchen for food preparation [AOR:8.59,Cl 95% (2.084–35.376)]. In other previous studies, mothers having a separate kitchen were identified as a significant factor which stated mothers who have separate kitchens might have protected complementary food from contaminated pathogens and that makes safe food for under two years old children so that these all help them keep good complementary food preparation practice [40,41]. This could be an understanding of good food hygiene handling practice with the availability of separate kitchen room safety packages.

### Limitations of the study

The study acknowledged some important possible limitations that should be considered when interpreting the results. First, the study was cross-sectional, a design that does not permit establishing cause-effect relationships. Second, social desirability and recall bias might be introduced.

## Conclusions

Our findings have indicated that hygienic practice of complementary food preparation among mothers with 6–24 months old children was poor. Age of women, partners’ educational status, having a modern type of stove, having a separated kitchen, and having a three bowls (utensils) system for complementary food preparation were factors, which significantly influenced the hygienic practice of complementary food preparation among women with children who were 6–24 months old. Therefore, it is crucial to address the problem under study with multidisciplinary strategies targeting on health education such as complementary food hygiene and safety and treatment of drinking water along value chain. Specifically, mothers or caregivers should wash their hands before preparing and feeding the complementary food for 6–24 months of age children, and a household should have a clean kitchen room separated from main house. Mothers should also have a three bowl dishwashing system for hygiene of complementary food preparation utensils. Thoroughly reheating any cooked, stored food before serving to children and washing hands with soap before serving cooked foods and feeding the children are also recommended.

## Supporting information

S1 Fig Knowledge Attitude and Hygiene practice during complementary food preparation among mothers with 6–24 months aged of children in debark town northwest, Etiopia,2021 (n = 423). (DOCX)

S1 Dataset (SPV)

S1 Annex English version questionnaire. (DOCX)

S2 Annex Amharic version questionnaire. (DOCX)

## Abbreviations

AOR: Adjusted Odds Ratio
CF: Complementary Feeding
COR: Crude Odds Ratio
ETB: Ethiopian Birr
HEW: Health Extension Worker
HHs: Households
HW: Hand Wash
WHO: World Health Organization

## References

[1] Organization, W.H., Complementary feeding: report of the global consultation, and summary of guiding principles for complementary feeding of the breastfed child. report of the global consultation, 2003.

[2] Gautam O., Food hygiene intervention to improve food hygiene behaviours, and reduce food contamination in Nepal: an exploratory trial. 2015, London School of Hygiene & Tropical Medicine.

[3] Jones AD I.S., Smith LE, et al, World Health Organization infant and young child feeding indicators and their associations with child anthropometry: a synthesis of recent findings Matern Child Nutr doi: 10.1111/mcn.12070, 2014. 10(1):1–17. 23945347 PMC6860255

[4] Samady W., et al., Recommendations on complementary food introduction among pediatric practitioners. JAMA network open, 2020. 3(8): p. e2013070–e2013070. 32804213 10.1001/jamanetworkopen.2020.13070 PMC7431991

[5] Das S., et al., Not water, sanitation and hygiene practice, but timing of stunting is associated with recovery from stunting at 24 months: results from a multi-country birth cohort study. Public health nutrition, 2020: p. 1–10. 10.1017/S136898002000004X PMC8025093 32404220

[6] Gizaw Z., Woldu W., and Bitew B.D., Child feeding practices and diarrheal disease among children less than two years of age of the nomadic people in Hadaleala District, Afar Region, Northeast Ethiopia. International breastfeeding journal, 2017. 12(1): p. 1–10. 10.1186/s13006-017-0115-z PMC5460459 28592985

[7] Shati A.A., et al., Occurrence of Diarrhea and Feeding Practices among Children below Two Years of Age in Southwestern Saudi Arabia. International journal of environmental research and public health, 2020. 17(3): p. 722. 31979127 10.3390/ijerph17030722 PMC7036833

[8] Ehiri J.E., et al., Critical control points of complementary food preparation and handling in eastern Nigeria. Bulletin of the World Health Organization, 2001. 79: p. 423–433. 11417038 PMC2566429

[9] Mattioli M.C., et al., Hands and water as vectors of diarrheal pathogens in Bagamoyo, Tanzania. Environmental science & technology, 2013. 47(1): p. 355–363. 10.1021/es303878d 23181394

[10] Oluwafemi F. and Ibeh I.N., Microbial contamination of seven major weaning foods in Nigeria. Journal of health, population, and nutrition, 2011. 29(4): p. 415. 10.3329/jhpn.v29i4.8459 PMC3190373 21957681

[11] Ahmed T., et al., Mortality in severely malnourished children with diarrhoea and use of a standardised management protocol. The Lancet, 1999. 353(9168): p. 1919–1922. 10.1016/S0140-6736(98)07499-6 10371570

[12] Derso T., et al., Stunting, wasting and associated factors among children aged 6–24 months in Dabat health and demographic surveillance system site: A community based cross-sectional study in Ethiopia. BMC pediatrics, 2017. 17(1): p. 1–9. 28376746 10.1186/s12887-017-0848-2 PMC5379504

[13] Curtis V., et al., Hygiene: new hopes, new horizons. The Lancet infectious diseases, 2011. 11(4): p. 312–321. 21453872 10.1016/S1473-3099(10)70224-3 PMC7106354

[14] Cami Moss T.H.B., Mihretab Melesse Salasibew, Joanna Sturgess, Girmay Ayana, Desalegn Kuche, Sustainable Undernutrition Reduction in Ethiopia (SURE) evaluation study: a protocol to evaluate impact, process and context of a large-scale integrated health and agriculture programme to improvecomplementary feeding in Ethiopia. BMJ Open. doi: 10.1136/bmjopen-2018-022028, 2018. 30030320 PMC6059290

[15] Arikpo D., et al., Educational interventions for improving primary caregiver complementary feeding practices for children aged 24 months and under. Cochrane database of systematic reviews, 2018(5). 10.1002/14651858.CD011768.pub2 PMC6494551 29775501

[16] Byrd K., et al., Differences in complementary feeding practices within the context of the wash benefits randomized, controlled trial of nutrition, water, sanitation, and hygiene interventions in rural Kenya. The FASEB Journal, 2017. 31: p. 165.1–165.1.

[17] Agustina R., Satroamidjojo T.P.S., S., Boveeoudenhoven I. M. J., Feskens E. J. M., and Kok F. J., Association of food-hygiene practices and diarrhea prevalence among Indonesian young children from low socioeconomic urban areas. BMC Public Health 2013. vol. 13, no. 1 10.1186/1471-2458-13-977 PMC3813984 24138899

[18] Abdurahman A.A., et al., Magnitude and determinants of complementary feeding practices in Ethiopia: A systematic review and meta-analysis. Heliyon, 2019. 5(7): p. e01865. 31317077 10.1016/j.heliyon.2019.e01865 PMC6611936

[19] Ntaji M., Oyibo P., and Bamidele J., Food hygiene practices of mothers of under-fives and prevalence of diarrhoea in their children in Malawi. Journal of Medicine and Biomedical Research, 2014. 13(2): p. 134–145.

[20] Jee Hyun Rah A.A.C., Bhupendra Badgaiyan, Victor M Aguayo, and Suzanne Coates S.A., Household sanitation and personal hygiene practices are associated with child stunting in rural India: a cross-sectional analysis of surveys. BMJ Open:e005180. doi: 10.1136/bmjopen-2014-005180, 2015. 5. 25678539 PMC4330332

[21] Rockville M., USA: EPHI *Ethiopia Mini Demographic and Health Survey* Ethiopian Public Health Institute (EPHI) [Ethiopia] 2019.

[22] Zongrone A W.K., Menon P., Infant and young child feeding practices and child undernutrition in Bangladesh: insights from nationally representative data Public Health Nutr [Internet]. Available from: http://www.ncbi.nlm.nih.gov/pubmed/22564370, 2012. 15:4: p. 1697–70. 10.1017/S1368980012001073 22564370

[23] Mitchodigni I.M., et al., Complementary feeding practices: determinants of dietary diversity and meal frequency among children aged 6–23 months in Southern Benin. Food Security, 2017. 9(5): p. 1117–1130.

[24] Mohammed S. and Tamiru D., The burden of diarrheal diseases among children under five years of age in Arba Minch District, southern Ethiopia, and associated risk factors: a cross-sectional study. International scholarly research notices, 2014. 2014. 10.1155/2014/654901 PMC4897213 27433486

[25] Semira Manaseki-Holland B.M., Karla Hemming, Martin James T., Christopher Bradley, Jackso Louise, etail, Effects on childhood infections of promoting safe and hygienic complementary-food handling practices through a communitybased programme: A cluster randomised controlled trial in a rural area of The Gambia. PLoS Med: 10.1371/journal. pmed.1003260, 2021. 18(1). PMC7799804 33428636

[26] Agustina R S.T., Satroamidjojo S, Ingeborg MJ, et al, Association of food-hygiene practices and diarrhea prevalence among Indonesian young children from low socioeconomic urban areas BMC Public Health, vol., 2012. 13, 977. 10.1186/1471-2458-13-977 PMC3813984 24138899

[27] Lidiya Dagne Asmare M.W.K., Ayele Mamo Abebe, Biruk Beletew Abate, Kirubel Dagnaw Tegegne, Prevalence and Factors Associated with Child Feeding Practice Among Mothers of Woldia Town, Northeast Ethiopia. Dove Press journal: Nutrition and Dietary Supplements, 2021. 12 p. 205–213.

[28] Demmelash A.A.M., Biruk Demissie Admasu, Fitalew Tadele, Bayih Eniyew, Tegegne Yitbarek, Getachew Yideg, Hygienic Practice during Complementary Feeding and Associated Factors among Mothers of Children Aged 6–24 Months in Bahir Dar Zuria District, Northwest Ethiopia, 2019. Environmental and Public Health, 2020. 2020.

[29] Gebre Yitayih K.B., Mahlet Tsegaye, Assessment of Hygienic Practice on Complementary Food among Mothers with 6–24 Months Age Living Young Children in Mohoni Town, North Eastern Ethiopia, 2015. Journal of Immunology, 2015.

[30] Henok Dagne L.B., Muluneh Borcha, Anley Tesfayeand Baye Dagnew, Hand washing practice at critical times and its associated factors among mothers of under five children in Debark town, northwest Ethiopia, 2018. Italian Journal of Pediatrics 2019. 45:120. 31519187 10.1186/s13052-019-0713-z PMC6743165

[31] Alelign Alemu Demmelash B.D.M., Fitalew Tadele Admasu, Eniyew Tegegne Bayih, Getachew Yideg Yitbarek, Hygienic Practice during Complementary Feeding and Associated Factors among Mothers of Children Aged 6–24 Months in Bahir Dar Zuria District, *Northwest Ethiopia* Journal of Environmental and Public Health, 2019. 2020: p. 7.

[32] Desta Dugassa Fufa A.A., Awgichew Teshome, Kedir Teji, Fistum Abera, Maleda Tefera, etail, Hygienic Practice of Complementary Food Preparation and Associated Factors among Mothers with Children Aged from 6 to 24 Months in Rural Kebeles of Harari Region, Ethiopia. Food Science and Technology 2020. 8(2) p. 34–42.

[33] C.P B., Magnitude of hygienic practices of food preparation and its associated factors working as volunteers in selected rural kebeles of Puttarh District, *Northern Sudan* International food journal 2018. 22(6) p. 2650–2656.

[34] Akoma Okugn D.W., Food hygiene practices and its associated factors among model and non model households in Abobo district, southwestern Ethiopia: Comparative cross-sectional study. PLOS ONE | 10.1371/journal.pone.0194391, 2018: p. p. pp. 294–303. PMC5886398 29621267

[35] Ayehu Gashe Tessema K.A.G.a.D.H.C., Factors affecting food handling Practices among food handlers of Dangila town food and drink establishments, North West Ethiopia. BMC PUblic Health.http://www.biomedcentral.com/1471-2458/14/571, 2014. 15:14. 10.1186/1471-2458-14-571 PMC4057591 24908104

[36] Contzen N D.P.S., Mosler HJ, Over-reporting in handwashing self- reports: potential explanatory factors and alternative measurements. PLoS One): 10.1371/journal.pone.0136445 2015. 10(8(e0136445). 26301781 PMC4547747

[37] Babita Susan Kuruvilla S.C.R., Jenyz M Mundodan, Jini M.P, Assessment and comparison of hygiene practices on complementary feeds among mothers in the urban and rural area in Thrissur district, Kerala. Indian Journal of Forensic and Community Medicine, Apr-Jun 2019. 6(2):66–69.

[38] USAID, Behavior Profile: Complementary Feeding: Hygienic Food Preparation & Feeding. Improve maternal and child survival and reduce malnutrition.

[39] WHO, *Infant and young child feeding*. WHO, Geneva,Switzerland, 2020.

[40] Numeri Chalumpha Geresomo E.W.K.-M., Joseph Wafula Matofari, Agnes Mbachi Mwangwela, Child Feeding Practices and Factors (Risks) Associated with Provision of Complementary Foods Among Mothers of Children Age 6–23 Months in Dedza District of Central Malawi. https://www.researchgate.net/publication/331658856, 2017.

[41] Bhadra Y Trivedi S.N.V., Bhavin S Dave, Khyati A Desai, Complementary feeding practices among mothers of Waghodia Taluka of Vadodara: a knowledge, attitude, and practice study. International Journal of Medical Science and Public Health, 2015. Vol 4 (5).

