# Supplementary material for: Hygienic practice during complementary food preparation and associated factors among mothers of children aged 6–24 months in Debark town, northwest Ethiopia, 2021: An overlooked opportunity in the nutrition and health sectors
Source: PLoS One. 2022 Dec 9;17(12):e0275730. doi: 10.1371/journal.pone.0275730 (PMC9733846; doi:10.1371/journal.pone.0275730)
Supplement: S1 Annex — (DOCX) [file pone.0275730.s003.docx]

**Part I: Socio-demographic characteristics of mothers**

| **S/No** | **Questions** | **Answer** | **Skip** |
| --- | --- | --- | --- |
| 101 | Age of the mothers(years) | _________ |  |
| 102 | Ethnicity | 1. Amhara 2. Tigray 3. Others |  |
| 103 | Religion of mother/caregiver | 1. Orthodox 2. Muslim 3. protestant 4. catholic 5. Others____________ |  |
| 104 | Level of education of mother/caregiver | 1. Unable to read &write 2. Read and write 3. Primary level 4. Secondary level 5. Diploma and above |  |
| 105 | Maternal occupation | 1. Unemployed 2. Civil servant 3. Daily laborer 4. Merchant 5. housewife 6. Farmer 7. Other(specify)__________ |  |
| 106 | Marital status of mother/caregiver | 1. Single 2. Married 3. Divorced 4. Widowed 5. lives separately |  |
| 107 | If you have a husband, what is the educational level of your husband? | 1. Unable to read &write 2. Read and write 3. Primary level 4. Secondary level 5. Diploma and above |  |
| 108 | If you have a husband, what is the occupational status of your husband? | 1. Unemployed 2. Civil servant 3. Daily laborer 4. Merchant 5. Farmer 6. Other specify ___________ |  |
| 109 | Total number of family size | _______________ |  |
| 110 | Total number of <2 years children | _______________ |  |
| 111 | Household average monthly income | _______________ |  |
| 112 | Access to media (TV or radio) | 1. Yes 2. No |  |
| 113 | Did you get children’s food preparation related training | 1. Yes 2. No |  |

**Part II: Household and environmental related factors**

| **S/No** | **Questions** | **Answer** | **skip** |
| --- | --- | --- | --- |
|  | About latrine conditions | |  |
| 201 | Do you have latrine for the household members? | 1. Yes 2. No | If 201=2, skip to 206 |
| 202 | Observe that which type of latrine the household have? | 1. pour flush latrine 2. Ventilated improved pit latrine (VIP) 3. Pit latrine with slab 4. Pit latrine without slab/open pit 5. No facilities or bush or field |  |
| 203 | Is the latrine serves single household? | 1. Yes 2. No |  |
| 204 | Is there hand washing facility near the latrine? | 1. Yes 2. No | If 203=2, skip to 206 |
| 205 | If the answer for Q 403 is yes, does the container have water? | 1. Yes 2. No |  |
| 206 | If the answer for Q 403 is yes, is there any type of soap or detergent with it? | 1. Yes 2. No |  |
| 207 | Do you wash your hands with soap after visiting toilets? | 1. Yes, regularly/always 2. Yes, sometimes 3. Never |  |
| 208 | Do you wash your hands with soap after cleaning child’s bottom? | 1. Yes, Always 2. Yes, but not always 3. Wash only with water 4. Never |  |
| 209 | Do you wash your child’s hands with soap after he/she defecates? | 1. Yes, Always 2. Yes, but not always 3. Wash only with water |  |
|  | About water supply | |  |
| 210 | Where do you get drinking water? | 1. Piped water 2. Protected well water 3. Protected spring water 4. Unprotected well water 5. Unprotected spring water 6. River water |  |
| 211 | How long does it take to go there, get water, and come back? | 1. Water source in the yard 2. Less than 30 minutes 3. Greater than 30 minutes |  |
| 212 | On average how much litter of water do your family use per day | **_____________________** |  |
| 213 | Household drinking water treatment options | 1. Chlorine 2. Wuha Agar 3. Boiling 4. No usage of treatment |  |
|  | Presence of separate kitchen and related appliances | |  |
| 214 | Is there separate kitchen for food preparation from the main house? | 1. Yes 2. No |  |
| 215 | Observe the type of cook stove in the kitchen the mother utilized. | 1. Cultural stove 2. Modern stove |  |
| 216 | Is there separate area to store raw and cooked foods? | 1. Yes 2. No |  |
| 217 | Is there three bowl system dishwashing facility in the kitchen? | 1. Yes 2. No |  |

Part III: Maternal knowledge about hygienic complementary preparation

| S/No. | Questions | Multiple Choice |
| --- | --- | --- |
| 301 | What is the best way to wash your hands when you are preparing food? | 1. Wash the hands with soap and water 2. Wash the hands with still water only 3. There is no need to wash hands if there is no any visible dirty. |
| 302 | Washing hands after changing a diaper | 1. Increases the chance of getting food borne diseases 2. Decreases the chance of getting food borne diseases 3. Makes no difference 4. The urine and feces of the child are safe and do not lead to food borne diseases |

**Food Safety Knowledge about “Keeping Clean” during complementary food preparation**

| S/No. |  | Correct code 1 incorrect code 0 | | |
| --- | --- | --- | --- | --- |
|  |  | Yes | No | I don’t know |
| 303 | To prepare safe food hands should be |  |  |  |
|  | 1. Properly cleaned |  |  |  |
|  | 1. Free of wounds |  |  |  |
|  | 1. With short and clean nails |  |  |  |
|  | 1. Unvarnished nails |  |  |  |
| 304 | It is not important to wash the child’s hands while eating |  |  |  |
| 305 | It is critical to wash hands: |  |  |  |
|  | 1. Before cooking |  |  |  |
|  | 1. Before serving meal |  |  |  |
|  | 1. Before taking meal |  |  |  |
|  | 1. Before feeding babies |  |  |  |
|  | 1. After visiting the toilet |  |  |  |
|  | 1. After changing baby’s diapers |  |  |  |
| 306 | Keeping raw and cooked food together does not lead to food contamination |  |  |  |
| 307 | Food handling should be avoided during a gastro intestinal illness (diarrhea, vomiting, etc). |  |  |  |
| 308 | It is not important to wash hands with soap after handling raw, beef, poultry or fish. |  |  |  |
| 309 | It is not important to store food in containers with lids in order to avoid contact between raw and prepared foods. |  |  |  |
| 310 | Leftover foods should be eaten without reheating them. |  |  |  |
| 311 | Contaminated water can be a source of food contamination |  |  |  |
| 312 | Fruits and vegetables should not be washed before eating. |  |  |  |

Part IV: Maternal Attitude about hygienic complementary food preparation

| S/No. | Questions | Multiple Choice |
| --- | --- | --- |
| 401 | The best way to wash your hand during food preparation is wash the hands with soap and water | 1. Strongly agree 2. Agree 3. Not applicable/undecided 4. Disagree 5. Strongly disagree |
| 402 | Washing hands after changing a diaper increases the chance of getting food borne diseases. | 1. Strongly agree 2. Agree 3. Not applicable/undecided 4. Disagree 5. Strongly disagree |
| 403 | Washing hands after changing a diaper decreases the chance of getting food borne diseases. | 1. Strongly agree 2. Agree 3. Not applicable/undecided 4. Disagree 5. Strongly disagree |
| 404 | Washing hands after changing a diaper makes no difference in food hygiene. | 1. Strongly agree 2. Agree 3. Not applicable/undecided 4. Disagree 5. Strongly disagree |
| 405 | The urine and feces of the child are safe and do not lead to food borne diseases. | 1. Strongly agree 2. Moderately agree 3. Agree 4. Moderately disagree 5. Disagree |
| 406 | To prepare safe food hands should be properly cleaned. | 1. Strongly agree 2. Moderately agree 3. Agree 4. Moderately disagree 5. Disagree |
| 407 | To prepare safe food hands should be free of wounds | 1. Strongly agree 2. Moderately agree 3. Agree 4. Moderately disagree 5. Disagree |
| 408 | To prepare safe food hands should be with short and clean nails | 1. Strongly agree 2. Moderately agree 3. Agree 4. Moderately disagree 5. Disagree |
| 409 | To prepare safe food hands should be unvarnished nails | 1. Strongly agree 2. Moderately agree 3. Agree 4. Moderately disagree 5. Disagree |
| 410 | It is not important to wash the child’s hands while eating. | 1. Strongly agree 2. Moderately agree 3. Agree 4. Moderately disagree 5. Disagree |
| 411 | It is critical to wash hands before cooking. | 1. Strongly agree 2. Moderately agree 3. Agree 4. Moderately disagree 5. Disagree |
| 412 | It is critical to wash hands before serving meal | 1. Strongly agree 2. Agree 3. Not applicable/undecided 4. Disagree 5. Strongly disagree |
| 413 | It is critical to wash hands before feeding babies | 1. Strongly agree 2. Agree 3. Not applicable/undecided 4. Disagree 5. Strongly disagree |
| 414 | It is critical to wash hands after visiting the toilet | 1. Strongly agree 2. Agree 3. Not applicable/undecided 4. Disagree 5. Strongly disagree |
| 415 | It is critical to wash hands after changing baby’s diapers | 1. Strongly agree 2. Agree 3. Not applicable/undecided 4. Disagree 5. Strongly disagree |
| 416 | Keeping raw and cooked food together does not lead to food contamination | 1. Strongly agree 2. Agree 3. Not applicable/undecided 4. Disagree 5. Strongly disagree |
| 417 | Food handling should be avoided during a gastro intestinal illness (diarrhea, vomiting, etc). | 1. Strongly agree 2. Agree 3. Not applicable/undecided 4. Disagree 5. Strongly disagree |
| 418 | It is not important to wash hands with soap after handling raw, beef, poultry or fish. | 1. Strongly agree 2. Agree 3. Not applicable/undecided 4. Disagree 5. Strongly disagree |
| 419 | It is not important to store food in containers with lids in order to avoid contact between raw and prepared foods. | 1. Strongly agree 2. Agree 3. Not applicable/undecided 4. Disagree 5. Strongly disagree |
| 420 | Leftover foods should be eaten without reheating them. | 1. Strongly agree 2. Agree 3. Not applicable/undecided 4. Disagree 5. Strongly disagree |
| 421 | Contaminated water can be a source of food contamination. | 1. Strongly agree 2. Agree 3. Not applicable/undecided 4. Disagree 5. Strongly disagree |
| 422 | Fruits and vegetables should not be washed before eating. | 1. Strongly agree 2. Agree 3. Not applicable/undecided 4. Disagree 5. Strongly disagree |

**Part IV: hygienic practices of mothers during complementary food preparation.**

| **S/No** | **Questions** | **Answer** | **skip** |
| --- | --- | --- | --- |
|  | Hygienic practices during food preparations and complementary feeding | |  |
| 501 | Do you wash your hands with soap before food preparation? | 1. Yes, Always 2. Yes, but not always 3. Wash only with water |  |
| 502 | Do you wash your hands with soap after touching raw foods and contaminated surfaces during food preparation? | 1. Yes, Always 2. Yes, but not always 3. Wash only with water |  |
| 503 | Does the utensils used to prepare the food are meticulously clean? Please check with observation. | 1. Yes 2. No |  |
| 504 | Do you wash your hands with soap before feeding the child? | 1. Yes, Always 2. Yes, but not always 3. Wash only with water |  |
| 505 | Do you wash your child’s hands with soap before eating/ feeding the child? | 1. Yes, Always 2. Yes, but not always 3. Wash only with water |  |
| 506 | Is there children’s private feeding utensil? | 1. Yes 2. No |  |
| 507 | Is the feeding utensil used to feed your child is a bottle? | 1. Yes 2. No |  |
| 508 | Does children’s private feeding utensils are clean? | 1. Yes (if they are washed thoroughly and look clean) 2. No (if utensils are dusty and look dirty) |  |
| 509 | Do you wash food utensils with soap? | 1. Yes, Always with soap 2. Yes, but not always with soap 3. Yes, Wash with ash 4. Wash with water only |  |
| 510 | Do you use of hot water for cleaning food utensils? | 1. Y es 2. No |  |
| 511 | Do you store raw and cooked food separately? | 1. Yes 2. No |  |
| 512 | Do you store utensils for raw and cooked food separately? | 1. Yes 2. No |  |
| 513 | Do you serve cooked food for children within 2 hours of preparation? | 1. Yes 2. No |  |
| 514 | Do you store ready-to-eat food in clean containers and cover it properly | 1. Always 2. Not always |  |
| 515 | Do you serve leftover food for children? | 1. Yes, always serve leftover food 2. Yes, sometimes serve leftover food 3. Never serve leftover food |  |
| 516 | Does the mother’s fingernail cut short? | 1. Yes 2. No |  |

Thank you for your valuable information and participations!!!
