# Supplementary material for: Hygienic practice during complementary food preparation and associated factors among mothers of children aged 6–24 months in Debark town, northwest Ethiopia, 2021: An overlooked opportunity in the nutrition and health sectors
Source: PLoS One. 2022 Dec 9;17(12):e0275730. doi: 10.1371/journal.pone.0275730 (PMC9733846; doi:10.1371/journal.pone.0275730)
Supplement: S2 Annex — (DOCX) [file pone.0275730.s004.docx]

**ክፍል 1፡የሶሽዮ-ዲሞግራፉመረጃመጠይቅ**

| ተ/ቁ | ጥያቄ | አማራጭ | እለፍ |
| --- | --- | --- | --- |
| 101 | የእናትየዋዕድሜበአመት | _________________ |  |
| 102 | የእናትየዋብሄር | 1. አማራ 2. ትግሬ 3. ሌላካላይገለፅ____________ |  |
| 103 | የቤተሰብወይምያአሳዳጊሀይማኖት | 1. ኦርቶዶክስ 2. ሙስሊም 3. ፕሮቴስታንት 4. ካቶሊክ 5. ሌላካለይገለፅ______________ |  |
| 104 | የሕፃኑ/ኗእናት/አሳዳጊየትምህርትደረጃ | 1. ማንበብናመፃፍየማትችል 2. ማንበብናመፃፍየምትችል 3. አንደኛደረጃ 4. ሁለተኛደረጃ 5. ኮሌጂናዩንቨርስቲ |  |
| 105 | የሕፃኑ/ኗእናት/አሳዳጊየስራሁኔታ | 1. ስራየሌላት 2. የመንግስትሰራተኛ 3. የቀንሰራተኛ 4. ነጋዴ 5. የቤትእመቤት 6. ሌላካለይገለፅ______________ |  |
| 106 | የሕፃኑ/ኗእናት/አሳዳጊየጋብቻሁኔታ | 1. ያላገባች 2. ያገባች 3. የፈታች 4. ባልየሞተባት |  |
| 107 | ባለቤትካለዎትየባለቤትዎየትምህርትደረጃ | 1. ማንበብናመፃፍየማይችል 2. ማንበብናመፃፍየሚችል 3. አንደኛደረጃ 4. ሁለተኛደረጃ 5. ዩንቨርስቲናኮሌጂ |  |
| 108 | ባለቤትካለዎትየባለቤትዎየስራሁኔታ | 1. ስራየሌለው 2. የመንግስትሰራተኛ 3. የቀንሰራተኛ 4. ነጋዴ 5. ገበሬ 6. ሌላካለይገለፅ_____________ |  |
| 109 | ጠቅላላየቤተሰብቁጥርብዛት | ________________ |  |
| 110 | ጠቅላላከ2 አመትበታችህፃናትብዛት | ________________ |  |
| 111 | የቤተሰቡአማካይወርሃዊገቢ | ________________ |  |
| 113 | የሚድያተደራሽነትአላቸው(ራዲዮ/ቴሌቭዥን) | 1. አዎን 2. የለም |  |
| 114 | ስለ ህፃናት ምግብ አዘጋጃጀት ስልጠና ወስደዉ ያዉቃሉ; | 1. አዎን 2. የለም |  |

**ክፍል2: የቤትእናየአካባቢሁኔታ**

| ተ/ቁ | ጥያቄዎች | | አማራጭ | | እለፍ |
| --- | --- | --- | --- | --- | --- |
|  | የመጸዳጃቤትንበተመለከተ | | | |  |
| 201 | ለቤተሰብዎየሚያገለግልየመፀዳጃቤትአለ? | | | 1. አዎን 2. የለም | If 201=2, skip to 206 |
| 202 | የመጸዳጃቤቱምንአይነትአንደሆነበምልከታይረጋገጥ | | | 1. በዉሃየሚሰራሽንትቤት 2. የአየርማስወጫቱቦያለዉሽንትቤት 3. ርብራብየሌለውባህላዊሽንትቤት 4. ርብራብያለውባህላዊሽንትቤት 5. ሽንትቤትየለም/ቁጥቋጦ/ሜዳላይ 6. ሌላካለይገለፅ___________ |  |
| 203 | መጸዳጃቤቱአገልግሎት የሚሰጠውለአንድየበተሰብአባላትብቻነው? | | | 1. አዎን 2. የለም |  |
| 204 | መጸዳጃቤቱአጠገብየተዘጋጀየእጅመታጠቢያአለ | | | 1. አዎን 2. የለም | If 203=2, skip to 206 |
| 205 | ለጥያቄቁ. 403 መልስዎአዎንከሆነ፣የእጅመታጠቢያውበውስጡውኃአለው | | | 1. አዎን 2. የለም |  |
| 206 | ለጥያቄቁ. 403 መልስዎአዎንከሆነ፣ለእጅመታጠቢያውአጠገብሳሙናወይምሌላየንፅህናመጠበቂያአለ? | | | 1. አዎን 2. የለም |  |
| 207 | መፀዳጃቤትከተጠቀሙበኃላእጅዎንበውኃእናበሳሙናይታጠባሉ? | | | 1. አወንዘወትር/ሁልጊዜ 2. አወንአልፎአልፎ 3. በውኃብቻእታጠባለሁ 4. አልታጠብም |  |
| 208 | ሕፃናትካፀዳዱበኃላእጅዎንበውኃእናበሳሙናይታጠባሉ? | | | 1. አወንዘወትር/ሁልጊዜ 2. አወንአልፎአልፎ 3. በውኃብቻእታጠባለሁ 4. አልታጠብም |  |
| 209 | ሕፃናትከተፀዳዱ/ካፀዳዱበኃላእጃቸውንበውኃእናበሳሙናያጥባሉ? | | | 1. አወንዘወትር/ሁልጊዜ 2. አወንአልፎአልፎ 3. በውኃብቻእታጠባለሁ |  |
|  | የውኃአቅርቦትንበተመለከተ | | | |  |
| 210 | የቤተሰብዎአባላትበዋናነትየሚጠቀመውየውኃመገኛየትኛውነው? | | 1. የቧንቧውኃ 2. የተጠበቀየጉድጓድውኃ 3. የተጠበቀየምንጭውኃ 4. ያልጠበቀየጉድጓድውኃ 5. ያልተጠበቀየምንጭውኃ 6. የዝናብውኃ | |  |
| 211 | ወደመገኛውሂዶቀድቶለመመለስምንያህልጊዜይጨርሳል? | | 1. የውኃመገኛውበግቢውስጥነው 2. < 30 ደቂቃይጨርሳል 3. > 30 ደቂቃይጨርሳል | |  |
| 212 | ቤተስብዎበቀንበአማካይምንያህልሊትርውኃይጠቀማል? | | _________________ | |  |
| 213 | በቤትውስጥወኃንለማከምየሚጠቀሙትየትኛውንአማራጭይጠቀማሉ? | | 1. ክሎሪን 2. ውኃአጋር 3. ዉኃንማፍላት 4. ምንምአይነትህክምናአልጠቀምም | |  |
|  | የተለየ የምግብ ማብሰያ ቤትና ተያያዥ ነገሮች | | | |  |
| 214 | ከዋናውቤትየተለየየምገብማበሰያማድበትአለ? | 1. አለ 2. የለም | | |  |
| 215 | ምንአይነትማብሰያምድጃእንደሚጠቀሙበምልከታይረጋገጥ | 1. ባህላዊምድጃ 2. የተሻሻለምድጃ | | |  |
| 216 | ለበሰሉ ምግቦችና ለጥሬ ምግቦች የተለየ ማከማቻ/ማሰቀመጫ አለ? | 1. አለ 2. የለም | | |  |
| 217 | ባለ ሶስት ጉድጓድ የማድ ቤት እቃዎች ማጠቢያ የተዘጋጀ ቦታ አለ? | 1. አለ 2. የለም | | |  |

**ክፍል3.ተጨማሪ ምግብ ስለመመገብንጽህናየእናቶችዕውቀት**

| ተ.ቁ | ጥያቄ | ምርጫዎች |
| --- | --- | --- |
| 301 | ምግብበምታዘጋጅበትጊዜእጅንመታጠብየምትችለውከሁሉየተሻለውመንገድምንድንነው? | 1. እጆችንበሳሙናናበውሃመታጠብ  2. እጆቻቸውንበውሃብቻመታጠብ  3. የሚታይቆሻሻከሌለእጆችንመታጠብአያስፈልግም |
| 302 | ሽንት ጨርቅከተቀየሩበኋላእጃችንመታጠብ | 1. በምግብ ወለድ በሽታዎች የመያዝ እድሉን ይጨምራል  2. በምግብ ወለድ በሽታዎች የመጠቃቱን ዕድል ይቀንሳል  3. ምንም ለውጥ አያመጣም  4. የህጻኑ ሽንት ና የሽሙጥ በሽታ አስተማማኝ ከመሆኑም በላይ ለምግብ ወለድ በሽታዎች አይዳርግም |

**ተጨማሪ ምግብ በሚመግቡበትወቅትስለ "ንጽህናመጠበቅ" የምግብደህንነትዕውቀት**

| S/No |  | ትክክለኛኮድ 1 የተሳሳተኮድ 0 | | |
| --- | --- | --- | --- | --- |
|  |  | አዎ | አይደለም | አላውቀውም |
| 303 | ምግብን በጥሩ ሁኔታማዘጋጀትእጆችንምን መሆንአለበት |  |  |  |
|  | 1.በአግባቡማጽዳት |  |  |  |
|  | 2.ከቁስልነጻማድርግ |  |  |  |
|  | 3.አጫጭርእናንጹህጥፍሮች |  |  |  |
|  | 4.Unvarnished (ቀለም ያልተቀቡ) ጥፍሮች |  |  |  |
| 304 | ምግብሲበላየልጁንእጅማጠብአስፈላጊአይደለም |  |  |  |
| 305 | እጅመታጠብወሳኝየሚሆነው |  |  |  |
|  | ሀ.ምግብከማብሰልበፊት  ለ.ምግብከመመገብበፊት |  |  |  |
|  | ሐ. ምግብከመብላትበፊት |  |  |  |
|  | መ. ህጻናትንከመመገብበፊት |  |  |  |
|  | ሰ.ከመጸዳጃቤትከተመለሱበኋላ  ር.የህጻንሽንትከተቀየሩበኋላ |  |  |  |
| 306 | ጥሬእናየበሰሉምግቦችንአንድላይማስቀመጥየምግብብክለትንአያስከትልም |  |  |  |
| 307 | በጨጓራህመምጊዜ (ተቅማጥ, ማስመለስወዘተ) የምግብአያያዝንማስወገድይገባል |  |  |  |
| 308 | ጥሬ፣የበሬ፣የዶሮእርባታወይምዓሣከያዛችሁበኋላእጅንበሳሙናመታጠብአስፈላጊአይደለም |  |  |  |
| 309 | በጥሬናበበሰሉምግቦችመካከልእንዳይገናኙምግብንበዕቃመያዣዎችውስጥማስቀመጥአስፈላጊአይደለም |  |  |  |
| 310 | የተረፈውንምግብሳያሞቁማብላትይኖርባቸዋል |  |  |  |
| 311 | የተበከለውኃየምግብብክለትምንጭሊሆንይችላል |  |  |  |
| 312 | ከመብላትበፊትፍራፍሬዎችናአትክልቶችመታጠብየለባቸውም |  |  |  |

**ክፍል4.ተጨማሪ ምግብ ስለመመገብንጽህና የእናቶች አመለካከት**

| ተ.ቁ | ጥያቄ | ምርጫዎች |
| --- | --- | --- |
| 401 | ምግብ በምታዘጋጅበት ጊዜ እጅን መታጠብ የምትችለው ከሁሉ የተሻለው መንገድ ነዉ ብለዉ ይስማማሉ? | 1. በጣም እስማማለሁ 2. በመጠኑ እስማማለሁ 3. እርግጠኛ አደለሁም 4. በመጠኑ አልስማማም 5. በጣምአልስማማም |
| 402 | ሽንት ጨርቅ ከተቀየሩ በኋላ እጃችን መታጠብ በምግብ የሚመጣ በሽታን እንደሚጨምር ይስማማሉ? | 1. በጣም እስማማለሁ 2. በመጠኑ እስማማለሁ 3. እርግጠኛ አደለሁም 4. በመጠኑ አልስማማም 5. በጣምአልስማማም |
| 403 | ሽንት ጨርቅ ከተቀየሩ በኋላ እጃችን መታጠብ በምግብ የሚመጣ በሽታን እንደሚቀንስ ይስማማሉ? | 1. በጣም እስማማለሁ 2. በመጠኑ እስማማለሁ 3. እርግጠኛ አደለሁም 4. በመጠኑ አልስማማም 5. በጣምአልስማማም |
| 404 | ሽንት ጨርቅ ከተቀየሩ በኋላ እጃችን መታጠብ በምግብ የሚመጣ በሽታን ምንም እንደማያገኛኘዉ ይስማማሉ? | 1. በጣም እስማማለሁ 2. በመጠኑ እስማማለሁ 3. እርግጠኛ አደለሁም 4. በመጠኑ አልስማማም 5. በጣምአልስማማም |
| 405 | የህፃን ሽንትና ሰገራ የምግብ ወለድ በሽታ እንደማያመጣ ይስማማሉ ? | 1. በጣም እስማማለሁ 2. በመጠኑ እስማማለሁ 3. እርግጠኛ አደለሁም 4. በመጠኑ አልስማማም 5. በጣምአልስማማም |
| 406 | ጥሬ እና የበሰሉ ምግቦችን አንድ ላይ ማስቀመጥ የምግብ ብክለትን አያስከትልም ብልዉ ያምናሉ | 1. በጣም እስማማለሁ 2. በመጠኑ እስማማለሁ 3. እርግጠኛ አደለሁም 4. በመጠኑ አልስማማም 5. በጣምአልስማማም |

**ክፍል 4 ተጨማሪ ምግብ ስለመመገብንጽህና የእናቶች አመለካከት**

| ተ.ቁ | ጥያቄ | ምርጫዎች |
| --- | --- | --- |
| 407 | ምግብ ለማዘጋጀት እጃችን ንፁህ መሆን እንዳለበት ይስማማሉ | 1. በጣም እስማማለሁ 2. በመጠኑ እስማማለሁ 3. እርግጠኛ አደለሁም 4. በመጠኑ አልስማማም 5. በጣምአልስማማም |
| 408 | ምግብ ለማዘጋጀት እጃችን ላይ ቁስል የለለበት መሆን እንደለለበት ይስማማሉ | 1. በጣም እስማማለሁ 2. በመጠኑ እስማማለሁ 3. እርግጠኛ አደለሁም 4. በመጠኑ አልስማማም 5. በጣምአልስማማም |
| 409 | ምግብ በሚያዘጋጁት ወቅት የእጃችን ጥፍር ንፁህ እና አጭር መሆን እንዳለበት ይስማማሉ? | 1. በጣም እስማማለሁ 2. በመጠኑ እስማማለሁ 3. እርግጠኛ አደለሁም 4. በመጠኑ አልስማማም 5. በጣምአልስማማም ም |
| 410 | ምግብ በሚያዘጋጁት ወቅት የእጃችን ጥፍር ንፁህ መሆን የለበትም ብለዉ ይስማማሉ ? | 1. በጣም እስማማለሁ 2. በመጠኑ እስማማለሁ 3. እርግጠኛ አደለሁም 4. በመጠኑ አልስማማም 5. በጣምአልስማማም |
| 411 | ህፃናት ለመመገብ እጃቸዉን መታጠብ አይጠቅምም ብለዉ ይስማማሉ ? | 1. በጣም እስማማለሁ 2. በመጠኑ እስማማለሁ 3. እርግጠኛ አደለሁም 4. በመጠኑ አልስማማም 5. በጣምአልስማማም |
| 412 | መግብ ለማዘጋጀት እጅ መታጠብ በጣም አስፈላጊ ነዉ ብለዉ ይስማማሉ?. | 1. በጣም እስማማለሁ 2. በመጠኑ እስማማለሁ 3. እርግጠኛ አደለሁም 4. በመጠኑ አልስማማም 5. በጣምአልስማማም |
| 413 | ምግብ ከማቅረብ በፊት እጅ በጣም አስፈላጊ ነዉ ብለዉ ይስማማሉ.? | 1. በጣም እስማማለሁ 2. በመጠኑ እስማማለሁ 3. እርግጠኛ አደለሁም 4. በመጠኑ አልስማማም 5. በጣምአልስማማም |
| 414 | ህፃኑን መግብ ከመመገብዎ በፊት እጅ በጣም አስፈላጊ ነዉ ብለዉ ይስማማሉ? | 1. በጣም እስማማለሁ 2. በመጠኑ እስማማለሁ 3. እርግጠኛ አደለሁም 4. በመጠኑ አልስማማም 5. በጣምአልስማማም |
| 415 | ህፃኑን ሽንትቤት ካፀዳዱት በኀላ እጅ መታጠብ በጣም አስፈላጊ ነዉ ብለዉ ይስማማሉ? | 1. በጣም እስማማለሁ 2. በመጠኑ እስማማለሁ 3. እርግጠኛ አደለሁም 4. በመጠኑ አልስማማም 5. በጣምአልስማማም |
| 416 | የህፃኑን የሽንት ጨርቅ ከቀየሩ በኀላ እጅ መታጠብ በጣም አስፈላጊ ነዉ ብለዉ ይስማማሉ? | 1. በጣም እስማማለሁ 2. በመጠኑ እስማማለሁ 3. እርግጠኛ አደለሁም 4. በመጠኑ አልስማማም 5. በጣምአልስማማም |
| 417 | ጥሬ እና የበሰሉ ምግቦችን አንድ ላይ ማስቀመጥ የምግብ ብክለትን አያስከትልም ብልዉ ያምናሉ | 1. በጣም እስማማለሁ 2. በመጠኑ እስማማለሁ 3. እርግጠኛ አደለሁም 4. በመጠኑ አልስማማም 5. በጣምአልስማማም |
| 418 | የህፃኑን ምግብ ንፅህና መጠበቅ እንደ ተቅማጥን የመሳሰሉትን መከላከል እንደሚቻል ይስማማሉ? | 1. በጣም እስማማለሁ 2. በመጠኑ እስማማለሁ 3. እርግጠኛ አደለሁም 4. በመጠኑ አልስማማም 5. በጣምአ,ልስማማም |
| 419 | እንደ ስጋ የመሳሰሉትን ጥሬ ምግቦች ከነካኩ በኀላ እጅን በሳሙና መታጠብ አስፈላጊ አይደለም ብለዉ ይስማማሉ? | 1. በጣም እስማማለሁ 2. በመጠኑ እስማማለሁ 3. እርግጠኛ አደለሁም 4. በመጠኑ አልስማማም 5. በጣምአልስማማም |
| 420 | የህፃኑን ምግብ ክዳን ባለዉ እቃ ማስቀመጥ የምብ ንኪኪን መጠበቅ እንደማይቻል ይስማማሉ? | 1. በጣም እስማማለሁ 2. በመጠኑ እስማማለሁ 3. እርግጠኛ አደለሁም 4. በመጠኑ አልስማማም 5. በጣምአልስማማም |
| 421 | ተረፈ ምብቦችን እንደገና ሳያሞቁ መመገብ እንደሚቻል ይስማማሉ? | 1. በጣም እስማማለሁ 2. በመጠኑ እስማማለሁ 3. እርግጠኛ አደለሁም 4. በመጠኑ አልስማማም 5. በጣምአልስማማም |
| 422 | ዉሃ ዋነኛ የምግም መበከል ምክንያት እንደሆነ ይስማማሉ?. | 1. በጣም እስማማለሁ 2. በመጠኑ እስማማለሁ 3. እርግጠኛ አደለሁም 4. በመጠኑ አልስማማም 5. በጣምአልስማማም |
| 423 | ፍራፍሬ እና ቅጠላቅጠል ምግቦችን ከመመገዎ በፊት እጅን መታጠብ እንደለለበዎት ይስማማሉ?. | 1. በጣም እስማማለሁ 2. በመጠኑ እስማማለሁ 3. እርግጠኛ አደለሁም 4. በመጠኑ አልስማማም 5. በጣምአልስማማም |

**ክፍል 5፡ የእናትየው የንፅህና አጠባበቅ ትግበራን በተመለከተ**

| ተ/ቁ | ጥያቄዎች | አማራጭ | እለፍ |
| --- | --- | --- | --- |
|  | ምግብበሚዘጋጅበትጊዜየንፅህናሁኔታን በተመለከተ | |  |
| 501 | ምግብከማዘጋጀትዎበፊትእጅዎንበውኃእናበሳሙናይታጠባሉ? | 1. አወንዘወትር/ሁልጊዜ 2. አወንአልፎአልፎ 3. በውኃብቻእታጠባለሁ |  |
| 502 | ምግብበሚያዘጋጁበትጊዜያልበሰሉምግቦችንናየቆሸሹነገሮችንከነኩበኃላበፊትእጅዎንበውኃእናበሳሙናይታጠባሉ? | 1. አወንዘወትር/ሁልጊዜ 2. አወንአልፎአልፎ 3. በውኃብቻእታጠባለሁ |  |
| 503 | ምግብን ለማዘጋጀት የሚገለገሉባቸው አቃዎች ንፅህናቸው የተጠበቀ ነውን? በምልከታ ይረጋገጥ | 1. አወን 2. የለም |  |
| 504 | ሕፃናትከመመገብዎበፊትእጅዎንበውኃእናበሳሙናይታጠባሉ? | 1. አወንዘወትር/ሁልጊዜ 2. አወንአልፎአልፎ 3. በውኃብቻእታጠባለሁ |  |
| 505 | ሕፃናትምግብከመመገብዎበፊትእጃቸውንበውኃእናበሳሙናያጥባሉ? | 1. አወንዘወትር/ሁልጊዜ 2. አወንአልፎአልፎ 3. በውኃብቻእታጠባለሁ |  |
| 506 | ለሕፃናትብቻተለይቶየተዘጋጀየመመገቢያእቃአለ? | 1. አዎን 2. የለም |  |
| 507 | ሕፃናት ለመመገብ የሚጠቀሙበት የተለየ መመገቢያ እቃ ጡጦ ነውን? | 1. አዎን 2. የለም |  |
| 508 | ለሕፃናትብቻተለይቶየተዘጋጀየመመገቢያእቃንፅህናውየተጠበቀነው? | 1. አዎን(በደንብየታጠበናንፁህከሆነ) 2. የለም(አቧርያለውናሲታይየቆሸሸከሆነ |  |
| 509 | የመመገቢያእቃዎችንበውኃናበሳሙናያጥባሉ? | 1. አወንዘወትር/ሁልጊዜ 2. አወንአልፎአልፎ 3. አዎንአመድንበመጠቀም 4. በውኃብቻአጥባለሁ |  |
| 510 | የመመገቢያእቃዎችንለማጠብየሞቀውኃንይጠቀማሉ? | 1. አዎን 2. የለም |  |
| 511 | የበሰሉምግቦችናያልበሰሉምግቦችንለእየብቻያስቀምጣሉ? | 1. አዎን 2. የለም |  |
| 512 | የበሰሉምግቦችናያልበሰሉምግቦችንለማሰቀመጥየምንጠቀምባቸውንእቃዎችለእየብቻያስቀምጣሉ? | 1. አዎን 2. የለም |  |
| 513 | የበሰለምግብንከተዘጋጅበ2 ሰዓትውስጥለህፃናትይመግባሉ? | 1. አዎን 2. የለም |  |
| 514 | የበሰሉምግቦችንበንፁህእቃበደንብከድነውያሰቀምጣሉ? | 1. ሁልጊዜ 2. አንዳንዴ |  |
| 515 | የተረፈምግብንለሕፃናትይመግባሉ? | 1. አዎንሁልጊዜ 2. አዎንአልፎአልፎ 3. አልመግብም |  |
| 516 | የእናትየውየእጅጥፍርአጠርተደርጎየተቆረጠነው? | 1. አዎን 2. የለም |  |

**ስለሰጡኝ ጠቃሚ መረጃና ስለተሳትፎዎ ከልብ አመሰግናለሁ!!!**
