# Supplementary material for: Hygienic practice during complementary food preparation and associated factors among mothers of children aged 6–24 months in Debark town, northwest Ethiopia, 2021: An overlooked opportunity in the nutrition and health sectors
Source: PLoS One. 2022 Dec 9;17(12):e0275730. doi: 10.1371/journal.pone.0275730 (PMC9733846; doi:10.1371/journal.pone.0275730)
Supplement: Supplementary file 3 [file pone.0275730.s001.docx]

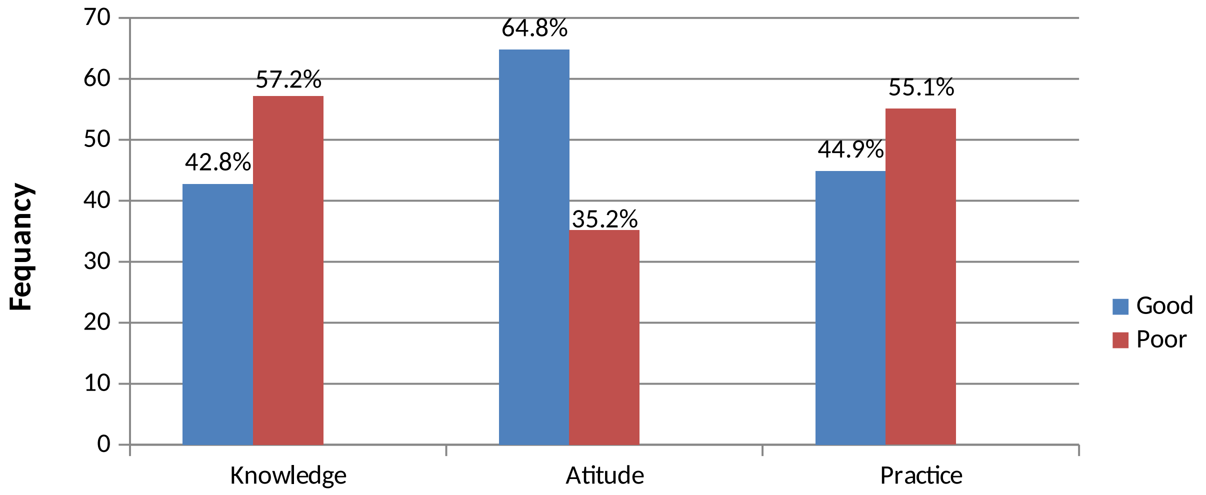

Figure: 1 knowledge,atitude and hygeiene practice during coplementary food preparation among mothers with 6-24 months aged of children in debark town Northwest ,Etiopia,2021 (n=423)
